# Thriving psychological well-being in undergraduate nursing student: a grounded theory study with the life grid approach

**DOI:** 10.1186/s12912-023-01338-1

**Published:** 2023-07-15

**Authors:** Lu Zhou, Thitinan Chankoson, YuMing Wu, EnLi Cai

**Affiliations:** 1 School of Nursing, Yunnan University of Chinese Medicine, Kunming, China; 2 Chakrabongse Bhuvanarth International Institute for Interdisciplinary Studies, Rajamangala University of Technology Tawan-OK, Bangkok, Thailand; 3 Faculty of Business Administration for Society, Srinakharinwirot University, Bangkok, Thailand; 4 School of Medicine, Yunnan University of Chinese Medicine, Kunming, China

**Keywords:** Life grid, Bachelor of nursing education, Mental health, Constructivist grounded theory, Symbolic interactionism

## Abstract

**Background:**

Psychological well-being (PWB) plays a vital role in successful adaptation to the Bachelor of Nursing journey and affects career development. However, there is little known about the functional and social processes associated with enhancing well-being specific to the subjective perspective of nursing students.

**Aim:**

To investigate how nursing students promote their psychological well-being to conceptualize thriving psychological well-being.

**Method:**

This qualitative study analyzed and reviewed a life grid and semi-structured in-depth interviews of 20 Chinese Nursing graduates by investigators and participants, following Charmaz's constructivist grounded theory. The constant comparative method was used to analyze data. This study took place between 2020 and 2022.

**Results:**

All participants experienced fluctuations in psychological well-being. This study identified a new understanding of how nursing students enhance their psychological well-being. Thriving awareness was co-constructed as the core category and based on the relationship with a supportive environment, the thriving psychological well-being of nursing students is conceptualized.

**Conclusions:**

It is imperative to enhance the psychological counseling and support for nursing students during their clinical placements, during the period just entering university as well as after repeated outbreaks of COVID-19. Nursing educators and administrators could develop appropriate educational programs and interventions based on the theoretical model—Thriving psychological well-being.

## Background

The mental health of nursing students has attracted considerable international attention [1, 2]. Although mental health issues and well-being are likely to be at opposite extremes of a continuum [3], enhancing nursing students' well-being may also lessen their psychological distress [4]. In the past few decades, "positive psychology" has also increased the attention of psychologists and other scientists to the concept of "well-being", implying different philosophical views. Diener E(1985) measured subjective well-being by Satisfaction with Life and emotional experience, under hedonic philosophy [5]. According to eudaimonic, the six components of psychological well-being (PWB) identified by Ryff (1989) are autonomy, environmental mastery, positive connections with others, purpose in life, the realization of potential, and self-acceptance [6]. According to Keyes (2002), well-being is formed when individuals display high amounts of hedonic and eudaimonic symptoms [7]. Seligman (2012) and Kern (2016), like Keyes, rely on both hedonic and eudaemonic philosophical foundations and suggested two models of well-being: the PERMA model (i.e. Positive emotion, engagement, relationships, meaning, and accomplishment) [8] and EPOCH model (i.e. engagement, perseverance, optimism, connectedness, and happiness) [9], respectively. Moreover, Feeney and Collins (2016) mentioned that closer interactions also increase PWB [10]. Evidence shows that greater well-being may be fostered by multidimensional components [11].

Psychological well-being was defined as the existence of psychological adjustment indicators as well as a sense of progress and self-realization [12, 13]. Research showed that psychological well-being is essential in nursing students' mental health as well as a successful adaptation to college/university life [14, 15]. Nursing students are more vulnerable to psychological distress than other professions due to the challenges encountered [16]. In China, the prevalence of anxiety, depression, and anxiety and depression co-occurring was, respectively, 55.0%, 56.4%, and 31.6% [17]. Psychological well-being also affects nursing students' decisions to stay in the nursing profession [18]. Meanwhile, a workforce shortage of nurses has been a major global concern, particularly during COVID-19 [19]. Around half (49.1%) of Chinese nursing students planned to switch majors, and nearly as many (45.4%) said they had no interest in becoming nurses after graduation [20]. Considering that poor PWB is associated with an increased likelihood of maladaptation and workforce shortages, it is crucial to understand how PWB is promoted among nursing students. Therefore, the main aim of the study was to investigate qualitatively the perspectives of undergraduate nursing students regarding aspects that promoted PWB along their journey.

Although extensive research has been carried out on the well-being of nursing students, the systematic mechanism in helping to promote psychological well-being among nursing students remains unknown. A review of the literature reveals that the available evidence has tended to focus on factors that reduced PWB or investigated indicators of psychological distress (e.g. [21, 22]). For example, considerable research on stress [23]; anxiety [24]; burnout [25]; and depression [26] has been gathered among nursing students. Furthermore, much of the research up to now has been quantitative, focused on correlation studies or hedonic well-being (e.g., subjective/emotional well-being). Social support [27], resilience [28], self-esteem [29], self-efficacy [30], and mindfulness [31] have been identified as being positively associated with nursing students’ well-being. While the existing evidence can help understand the link between PWB and these factors, it is equally valuable to explore the thriving mechanisms of psychological well-being, what promotes higher PWB in the undergraduate nursing program, to develop more holistic evidence of PWB in nursing students.

The presence of a research gap in the field indicated the need for the generation of a more comprehensive theoretical model for promoting PWB. This model would allow for the consolidation of knowledge in the area. Grounded theory [32] can be used to address this issue. To enrich the form of the data and to reduce recall bias, the life grid approach [33] was also conducted to improve the reliability of the results. This study aimed to construct a substantive theory of nursing students' perceived experience, knowledge, and skills for enhancing PWB based on a positive psychology perspective and conceptualize thriving psychological well-being.

## Methods

### Design

The paradigm for this qualitative investigation was Charmaz's (2014) constructivist grounded theory (CGT) methodology [32]. Researchers were encouraged to utilize their full interpretative abilities to delve into data collected in the field, collaborate with participants to form a theory, and then return to the field to test the theory's plausibility among study participants and other knowledge beneficiaries like nurses [32]. With the use of grounded theory, a substantive theory may be developed to better explain and visualize the core category, subcategories, and interrelationships of a specified substantive domain.

### Ethics approval

The studies involving human participants were reviewed and approved by the Ethics Committee, Rajamangala University of Technology Tawan-ok. Participants provided written informed consent and all survey materials were collected anonymously to protect confidentiality.

### Setting and participants

This study took place in southwest China, and twenty Bachelor of Nursing graduates 22–25 years of age (14 females and 6 males) from three universities in China (All full-time) took part. All participants earned their Baccalaureate by conducting an independent study, passing all program tests, and completing a clinical placement with a minimum duration of one year. Inclusion criteria required participants to complete data collection within two years of receiving confirmation of their bachelor's degree to aid recall.

### Recruitment and sampling

Social media posts were used to circulate information about the study (e.g. Wechat groups, Tencent Instant Messenger groups, and Sina Weibo). A non-probability sample of undergraduate nursing students was recruited from two universities in Southwest China using a purposeful sampling approach. Snowball sampling was also utilized. A total of 27 participants were enrolled in the study. Between July 2021 and February 2022, semi-structured in-depth interviews were conducted with 20 graduates. The remaining seven participants were not scheduled for interviews due to inclusion criteria, theoretical sampling, or personal reasons.

### Data collection

A semi-structured in-depth interview guide was developed by the lead author (ZL) and was employed to collect data with the life grid approach. The interviews were conducted via video-conferencing software (Zoom). After the sixteenth interview, data reached a level of saturation and no new findings emerged, four additional interviews were conducted to confirm saturation. The interviews varied in length between 60 and 150 min (average 105 min), and were audio-recorded and transcribed by a meeting record application. All participants received 10RMB as compensation for their time.

The life grid was designed with rows corresponding to each semester of each year during respondents’ undergraduate tenure, as well as two discrete periods, i.e., the beginning and the end of the bachelor's degree in nursing. The three indirect indicator columns included Bachelor's Nursing Journey, life Journey, and PWB. The grid spaces are intentionally left blank so that the interviewer and the interviewee can complete it collaboratively during the interview to enrich the qualitative interview data [34]. Initially, at the beginning of the interview, the life grid was presented and participants were prompted to build a well-being event timeline of major milestones (e.g., clinical placements) and other undergraduate nursing-related events that happened during their Bachelor of Nursing journey. The next step was for participants to report on any life changes they had experienced that had an impact on their PWB. Lastly, participants drew a free-form line from "low" to "high" to indicate how they felt about their PWB at various points along the route. During the interview, participants were encouraged to provide additional pertinent information.

A semi-structured in-depth interview approach was employed to enable participants to describe their thoughts and feelings throughout their undergraduate nursing journey. Based on feedback about psychological well-being on the life grid, several questions were asked of them to understand the factors they felt contributed to the 'high moments' of psychological well-being and a deeper understanding of these promoting factors (e.g. "Could you describe what contributed to your PWB at these moments?"). To achieve theoretical sampling, we also used probing questions and adapted interview questions.

### Data analysis

A visual inspection is formed by participants' feedback on their psychological well-being for each event in the life grid [35], visualizing fluctuations in psychological well-being with WebPlotDigitizer [36, 37].

Data analysis was performed simultaneously with data collection in accordance with CGT and the use of continuous comparison. Manual data analysis was utilized, including steps of initial, focused, and theoretical coding [32]. The researcher (ZL) immersed herself in the data, using the participants' words for in vivo coding whilst writing the memos and transcribing the interviews as she sought the meaning contained in the raw data. CGT assumes that the researcher has pre-existing knowledge and expertise in the field of study [38], contributing to the theoretical sensitivity. The researcher in this study has extensive experience in positive psychology and nursing education and acknowledges potential bias.

Line-by-line open coding was used initially [32]. Relevant concerns, events, and activities that may serve as a category were defined and coded, with each code representing context, situations, actions, interactions, and consequences [39]. Gerunds (verbs ending in 'ing') were employed widely to code transcripts. The capture of the use of gerunds allowed the research to focus more on the actions and processes rather than the individuals, facilitating the development of theoretical insights. Similar codes were grouped into categories, each defined by their properties and dimensions (Charmaz, 2014). Simultaneous data analysis and comparisons provided researchers with clues for subsequent interviews and guided theoretical sampling to facilitate subsequent stages of analysis. This stage was guided by Feeney and Collins' (2014) theoretical model of Thriving Through Relationships [40]. Focused coding was used to separate, classify and synthesize large data. This ensures data understanding, the development of emerging categories, and the co-constructed core or central phenomenon. Theoretical codes were used to conceptualize the connections between the codes. Data collection was discontinued when the 20th interview was theoretically saturated and no new codes were constructed. In addition to being utilized as a theoretical reference point for the development of the core category and the secondary category, these codes were also submitted to code constant comparison analysis, code with category comparison analysis, and category with category constant comparison analysis, as shown in Table 1.

**Table 1:** Thematic codes from the interview

Theoretical coding categories	Focused coding categories	Open coding categories	No. respondents mentioningeriod codes					
			**Reviewer 1**		**Reviewer 2**		**Reviewer 3**	
			**Sources**	**Codes**	**Sources**	**Codes**	**Sources**	**Codes**
**Thriving Awareness**	Perception and Appraisal of Events		17	130	16	106	19	118
		Epiphany and inspiration	12	38	9	21	14	33
		Savoring	12	50	15	41	15	62
		Self-efficacy	12	42	15	44	10	23
	Internal drive		20	123	22	95	21	129
		Contribution and sense of mission	9	17	10	14	9	23
		Faith	4	5	6	7	4	5
		Intrinsic rewards	15	42	13	33	15	41
		Self-expansion	17	59	20	41	17	60
	Self-representation		17	46	16	45	17	43
		Self-compassion	7	18	6	12	7	18
		Self-identify	15	28	15	33	13	25
	Situation-relevant Correspondences and Outcomes		20	118	22	101	21	110
		Accomplishments	19	78	19	66	19	73
		Resilience	8	16	8	13	8	16
		SOC	11	23	11	22	11	21
**Supportive Environment**	Social Support		20	111	20	110	20	110
		Family	7	16	7	16	7	15
		Schoolmate and peer	17	42	19	44	15	43
		Special others	13	22	11	19	13	23
		Supervisors	11	27	9	27	13	26
		Material support	4	4	5	4	4	3
	Relational Attitudes		16	43	15	41	10	35
		Attachment security	6	8	7	9	7	5
		Relational self-expansion	15	35	11	32	14	30
	Concentration		9	14	10	20	7	11
	Lifestyle		8	16	9	9	8	16

Being reflexive is important in CGT and theoretical memos were written to record the author’s thoughts, participants' nonverbal cues, theoretical questions, and coding summaries [41]. These were utilized to monitor and inspire more coding, as well as to serve as a foundation for theory integration and ultimate theory creation. This analysis was carried out in NVivo 11 Plus [42].

### Trustworthiness

Charmaz's (2006) criteria (i.e., credibility, resonance, originality, and usefulness) was used to increase the trustworthiness of the data. The credibility of this research was increased by using a reflective diary and according to the main tenets of grounded theory. Furthermore, the triangulation of data collection and analysis maintained the consistency of the conceptual categories as well as the variety of the sample. In this study, data were collected from a range of sources, from interviewees studying at different universities, over two recruitment methods. Triangulation was also achieved via team discussions of the data. Resonance was demonstrated using two approaches. Firstly, theoretical saturation was reached after interviewing 20 participants, whereby no new data or leads were arising and categories were sufficiently dense. Secondly, member checking was utilized [32]. Regarding originality, the interview outline, which was based on the life grid approach [33], made sure that new categories were explored, which led to new PWB-related insights. Moreover, the qualitative data analysis also yielded various new conceptual categories that might be used to develop the model. Regarding usefulness, the PWB enhancement procedure of nursing students was thoroughly defined to provide a theoretical foundation and practical implications for the enhancement of nursing students' mental health. The trustworthiness of the results was ensured by using a dispassionate approach to the interviews and a rigorous coding procedure. Specific strategies are detailed in Table 2.

**Table 2:** Guaranteeing strategies in terms of Criteria for the trustworthiness

Criteria	Strategies
**Credibility**	Data triangulation
	Member checking
	Negative case analysis
	Peer checking
	Memos
	Reflective diary
**Resonance**	Saturation
	Reflective diary
	Prolonged engagement
	Thick description
	Member checking
**Originality**	Theoretical sampling
	Use of life grid approach
	Use creative and flexible data analysis techniques to identify novel patterns and themes
**Usefulness**	Purposeful sampling
	Develop research findings that can be applied to practical contexts and have real-world relevance
	Dissemination

## Results

### Life grid approach

**Fig. 1:**
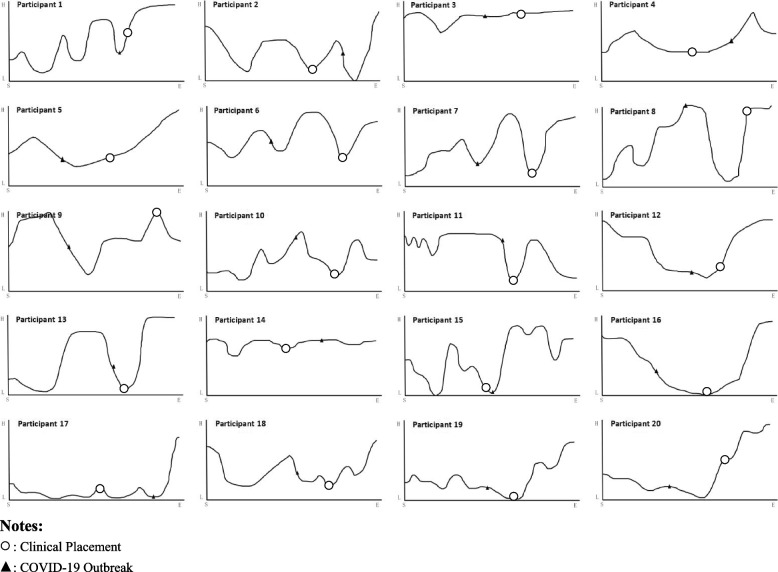
Fluctuations in psychological well-being for each participant formed through the life grid approach

All participants experienced “highs” and “lows” during the Bachelor of Nursing journey, with the high points typically reported during the mid and later stages of the Baccalaureate journey for most participants. The psychological well-being of nursing students fluctuated the most after the COVID-19 outbreak and clinical placements, as detailed in Fig. 1.

### Description of the concept

**Fig. 2:**
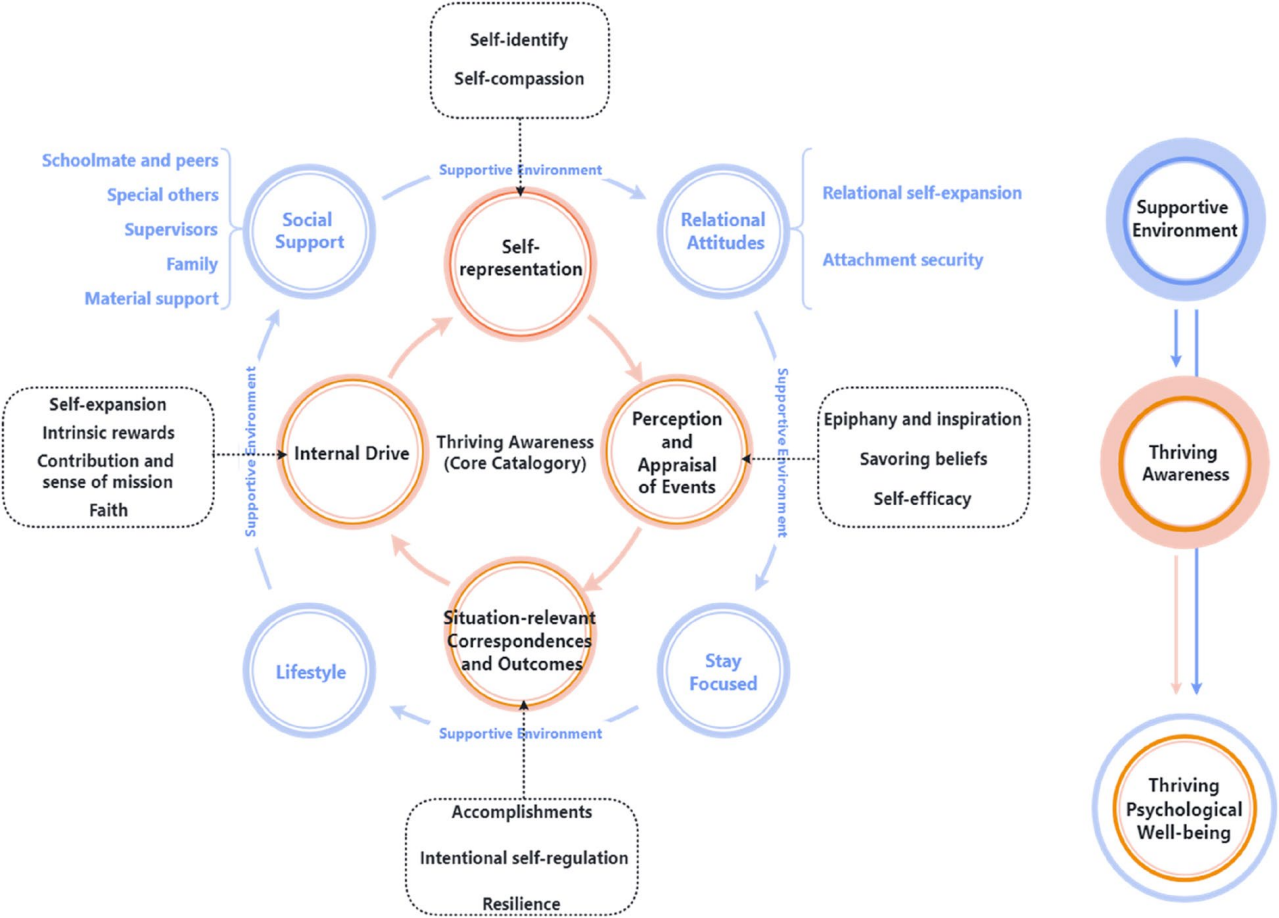
The model of thriving psychological well-being for undergraduate nursing students

The iterative process between data generation and analysis resulted in the development of a theoretical model. Two categories were co-constructed from 8 focused coding categories and 19 open coding categories: thriving awareness and supportive environment. Thriving awareness was co-constructed as the core category and based on the relationship with a supportive environment, which delineates conceptualizations of thriving psychological well-being among undergraduate nursing students, helped to promote PWB in the Baccalaureate Nursing journey, as illustrated in Fig. 2. Each category is presented in the following subsections.

#### Core category: ‘Thriving awareness’

The core category, ‘Thriving awareness’, is half the battle in promoting psychological well-being among undergraduate nursing students. This category represents an aggregate of the experiences that the nursing students went on as they constructed and adjusted their thoughts, knowledge, and willingness about the enhancement of psychological well-being over the university journey. There were 12 open coding categories and 4 focused coding categories leading to it directly.

- **Category 1:** Highs in PWB coincided with positive perception and appraisal of events along the journey. This category had three open coding categories:
- **Open coding category 1:** The upswing in PWB was associated with moments when participants derive *epiphany and inspiration*, started to feel that the nursing profession was “making sense” or figured out something.
- **Open coding category 2:** Participants regulated their positive feelings by directing their attention to positive experiences, appreciating these experiences, and elaborating on or enhancing the experience of these positive moments in their life. The upswing and highs of the PWB coincide with this ability, which is represented by *savoring beliefs*.
- **Open coding category 3:** Upswings and highs in PWB tended to match participants’ beliefs about their coping abilities to challenges, that is, s*elf-efficacy.*
- ***Category 2:*** The realization and satisfaction of an *internal drive* were also reported during positive phases of PWB, motivated to persevere and to stretch to new levels (not settle for good enough). This category had four open coding categories as follows:
- **Open coding category 4:** Participants reported that the enhancement in their PWB happened after participating in either the free clinic or pro bono activities. They were enjoying that *contribution and sense of mission*.
- **Open coding category 5: ***Faith* is the motivation to enhance PWB when it is in decline. Participants outlined that their faith in their profession, lives, etc., was instrumental in their PWB.
- **Open coding category 6: ***Intrinsic rewards* of inner fulfillment, autonomy, positive sensations, etc., contribute to the positive stimulation of PWB.
- **Open coding category 7:** The upswing in PWB is associated with moments when participants were engaged in novel, challenging, and exciting activities, and incorporating and absorbing others’ perspectives and experiences, represented b*y* s*elf-expansion.*
- ***Category 3:*** A crucial element depicted during positive periods of PWB was a continuous improvement of *self-representation*. This category had two open coding categories as follows:
- ***Open coding category 8:*** A better ability to endure negative events (e.g. Academic stress, workplace violence, negative perception of the professional image. etc.) and benevolence to oneself, that is, *self-compassion*, can maintain better PWB.
- ***Open coding category 9:**** Self-identity* and a positive professional self-concept play vital roles in enhancing nursing students' PWB.
- ***Category 4:*** Benign *situation-relevant correspondences and outcomes*, such as improvements in coping strategies, self-regulation, and production of high-quality results, are coincident with the high point of PWB in the undergraduate journey of nursing students. This category had three open coding categories as follows:
- ***Open coding category 10:*** Upswings in PWB matched *accomplishments* throughout the undergraduate journey of nursing. Beneficial outcomes included obtaining positive comments from advisors, getting good grades, progress in scientific research, nursing skills enhancement, etc.
- ***Open coding category 11:**** Resilience*, the ability of an individual to recover from a negative situation and adapt to the circumstances, produces greater PWB.
- ***Open coding category 12:**** Intentional self-regulation*, such as selection, optimization, and compensation of goals or tasks, enhances nursing students’ PWB.

#### Secondary category: ‘Supportive environment’

The secondary category, ‘*supportive environment’*, is the foundation of promoting psychological well-being. This category represents a haven and secure base support for the enhancement of psychological well-being. There were 7 open coding categories and 4 focused coding categories leading to it directly.

- ***Category 5:*** The significance of *social support* in negotiating the hurdles of the undergraduate experience was emphasized throughout the participant narratives. Highs in PWB were underpinned by support from different types. The open coding categories were:
- ***Open coding category 13–17:*** During moments of enhanced PWB, obtaining support from *schoolmates and peers(13), special others(14), supervisors(15), family(16),* and *material support(17)* was identified during moments of enhanced PWB. This wide support network was regarded to be the catalyst to share one’s experiences, during both adversity and prosperity.
- ***Category 6:*** Healthy *relational attitudes* are closely tied to promoting PWB, also as the social support catalyst. The open coding categories were:
- ***Open coding category 18:*** Participants with high trust and quality of close relationships, due to *attachment security*, experience good psychological outcomes.
- ***Open coding category 19:*** Upswings in PWB are frequently associated with acquiring and absorbing the attributes of a partner in a close relationship (e.g. Best friends, supervisors, parents, etc.), represented by *relational self-expansion*.
- ***Category 7:*** For participants who focus on the present moment, such as *staying focused* on the nursing program curriculum and their journey, it helps to promote positive PWB changes, closely tied to two other open coding categories, *intrinsic rewards,* and *accomplishments.*
- ***Category 8:*** A healthy ***lifestyle*,** such as maintaining a good study-life balance, increased physical and mental activity, and a healthier diet, was a final supportive element noted during positive periods of PWB.
- Overall, Participants’ understanding of thriving psychological well-being was detailed in Table 3.

**Table 3:** Narrative examples of categories within the data set

Categories	Narrative examples
Perception and Appraisal of Events	
Epiphany and inspiration	"I just feel that I am still inspired to have some new perspectives on this profession."-P8
Savoring	"Writing a diary during the clinical placement is actually a good way to record and remember, which gives me the strength to keep going."-P10
Self-efficacy	"Although I don't like the nursing profession right now, I think I'll grow to like it over these four years and I think I can do well with it."-P1
Internal drive	
Contribution and sense of mission	"Participating in community clinics as well as AIDS prevention charity activities, etc., is doing something like, just not knowing what's good for anyone, but doing something meaningful."-P20
Faith	"The faith in this profession makes me feel that since I have chosen to nurse, I am able to serve this health in the future, even if I don't work in this field in the future, but I still have to learn it solidly when I do."-P13
Intrinsic rewards	"I particularly enjoyed my clinical placement in a department with a good atmosphere; the division of tasks in the department was also particularly well-defined and I felt autonomous."-P12
Self-expansion	"I've grown quickly in these four years, and at every stage, I was the one who tried what I thought was impossible as well as what was a novelty."-P4
Self-representation	
Self-compassion	"It's just a self-assurance that everyone thought a final review was hard and it wasn't just me. So I will let myself take my time and not rush."-P9
Self-identify	"After participating in a nursing research group, I took it upon myself to carry out, something like a literature search, and for the first time, I felt a sense of identity and pride in the profession."-P15
Situation-relevant Correspondences and Outcomes	
Accomplishments	"The Neurological Intensive Care Unit was one of the departments where I gained the most. For example, my nursing skills have gotten a qualitative leap, and I remember that the first IV Catheter and the first aspiration of my life were completed in that department, so I feel a sense of achievement."-P19
Resilience	"With the death of the patient, and the influence of COVID-19, I feel a heavy workload, but I think I'm pretty resilient and will self-regulate."-P11
Intentional self-regulation	"I had a foolproof plan for student associations and studies because there were a lot of major courses offered in my junior year; I dropped all the associations because I wanted to focus on one thing, that is, one thing at a time."-P7
Social Support	
Family	"When I encountered setbacks and difficulties in my studies and life, my parents would encourage me and give me advice, reassuring me that ' to cross that bridge when I come to it'."-P4
Schoolmate and peer	"When I was very tired from my internship, chatting with my friends, I would feel a force that just supported me."-P16
Special others	"That time was actually in a very intense revision rushing stage. It was a cold winter day, I was studying on campus, and then a strange girl suddenly appeared in front of me and gave me a few candies, and then told me to cheer up for my final exam."-P3
Supervisors	"My mentor's professional help and advice helped to dispel my fears and self-doubt during my clinical placement."-P2
Material support	"No financial worries; Get a scholarship"-P3, P18
Relational Attitudes	
Attachment security	"I could share everything with my father and I would not feel bored at home during the COVID-19 outbreak. Actually, I was very comfortable and relaxed at home during the quarantine."-P6
Relational self-expansion	"I was deeply influenced by the personality of my surgical nursing lecturer. Those insights and interaction with others, I would learn and imitate in my mind."-P17
Concentration	"When I'm very concentrated on learning, it's very efficient and I also feel like, that' s awesome."-P14
Lifestyle	"Being close to nature and exercising regularly will make me feel comfortable and relaxed, like more optimistic."-P5

*P* Participant

## Discussion

This constructivist GT provides a theoretical perspective on how Chinese undergraduate nursing students promote their psychological well-being to conceptualize the thriving PWB. This study qualitatively explored undergraduate nursing students' perspectives of PWB-enhancing elements. The insights concentrate on the positive aspects of nursing students' experiences and provide extensive descriptions of the elements that promote PWB by referencing positive psychology and collecting data using LGM and interviews. These holistic and multifaceted sets of promoting elements play a vital role in strategies for mental health among nursing students.

Consistent with conceptualizations of PWB [8, 9, 13], positive relationships within the academic and professional community appeared to be instrumental to nursing students’ PWB, especially positive feedback from supervisors, patients, and peers. As regards relationships with patients, encouragement is vital to developing confidence and self-identity. Educational interventions that optimize the capability of dealing with the relationships with patients could be valuable [43], which better enables nursing students to adapt to changes in the academic-clinical environment. Interventions that target to manipulate and enhance other social support are also necessary [15]. As regards support from supervisors, alongside constructive and professional feedback, almost participants emphasized the benefits of care, especially during the clinical placement. Hence, the emphasis should be placed on programs that promote the growth of positive supervisor-student interactions. Universities might, for example, provide clinical and academic supervisors with preparation on the qualities of a helpful supervisory relationship and make ensuring that supervisors have enough time and experience to engage nursing students [37].

Echoing previous theories suggesting that social support is also effective in facilitating nursing students to draw on the strengths of their supervisors and other relationships [10]. Receiving support could lead to immediate relational benefits including feeling valued and appreciated, being able to pursue aspirations on one's own, experiencing new things, and self-expanding with a close friend [44].

Interestingly, the findings show that social support not only influences relational attitudes but also promotes concentration and lifestyle. For example, participants reported that they were more able to stay focused and keep a healthy lifestyle with well-established social support. Social support promotes the individual's ability to self-regulate and focus on the present moment to effectively cope with stress and challenges (e.g. Reduced usage of alcohol, tobacco, or other addictive drugs to reduce anxiety; increased sleep quality; and better adherence to medical regimens) [45]. Given that maintaining a healthy lifestyle is regarded as the responsibility of both nursing students and their institutions [46], we suggest that the transition period at the clinical placement of nursing programs, as well as during the COVID-19 pandemic, could provide a prime opportunity for institutions to intervene and encourage the adoption of such self-care practices. Thus, this could be able to assist nursing students in preventing mental health problems before they arise.

Social support can be regarded as the threshold for all facilitators, in line with the theory proposed by Feeney and Collins [10], with relational attitudes as the foundation, alongside concentration and lifestyle, forming a supportive environment for thriving PWB. Supportive environments also indirectly enhance psychological well-being via the core category/thriving awareness.

The participants highlight the importance of adaptation, response, and outcome to events and situations due to the benefits of promoting their PWB. Indeed, accomplishments, resilience, intentional self-regulation, epiphany and inspiration, savoring, and self-efficacy share theoretical intersections with conceptualizations of PWB, according to previous models including dimensions such as achievement [8], environmental mastery [13], and meaning [47]. Compared to non-medical peers, nursing students may experience a greater range of life challenges, including but not limited to exposure to death [48], workplace violence, negative portrayal of the nursing profession, and apprehension towards the COVID-19 pandemic [20, 49]. A greater ability to focus on positive experiences is the key to overcoming obstacles and challenges and predicted greater psychological well-being [50]. Specifically, nursing students' ability to better endure negative events and savor positive ones can promote self-acceptance and self-development, exert greater effort in tasks, and achieve greater accomplishments to maintain better psychological well-being [51]. Meanwhile, intentional self-regulation, such as selection, optimization, and compensation, can be used to deal with goal conflict and reach goals [52]. Additionally, a supportive environment predicts increased self-efficacy and perceived control to the extent that it has equipped the nursing students with courage, knowledge, resources, or skills to overcome the adverse circumstance [40, 53], that is, the supportive environment promotes processing, feedback, and adaptation to events and situations by enhancing the self-efficacy of nursing students.

Finally, the results demonstrated that internal drive and self-representation helped promote their PWB. Previous theories have suggested that support can encourage individuals to grow in the face of adversity, motivating them to change their lives, and work to rebuild and persevere through difficult times [54]. Conversely, in good times, a supportive environment can also motivate individuals to stretch to new levels and not settle for good enough, leaving one's comfort zone to grow and reach one's potential [10]. We extended the findings to Chinese nursing students and found that nursing students' internal drive is more likely to increase self-efficacy and self-concept clarity to enhance nursing students' professional self-concept and professional identity, thereby improving self-acceptance and self-growth to promote psychological well-being [15]. As such, we call for nursing students to be aware of the importance of intrinsic motivation to enhance their self-efficacy, as well as a sense of faith and mission in the nursing profession. Meanwhile, institutions could develop initiatives to promote nursing students' motivation and ability to explore novel, challenging, and exciting activities [55], as well as confidence in the nursing profession and nursing culture [56].

However, another noteworthy issue is that most of the nursing students interviewed have a negative self-perceived social identification with the nursing profession, which to some extent also affects their self-representation and self-integrity [57]. This, in turn, was the main reason for the significant decline in nursing students' psychological well-being upon entering clinical placements, as well as the period just entering university time. Therefore, it is also particularly crucial to educate nursing students about the mission of the nursing profession and nursing image [58]. Although previous studies have suggested that self-compassion is associated with increased self-identity and professional identity, very few nursing students interviewed were able to use self-compassion to promote psychological well-being. More fully, there are important differences in the modal cultures that exist in nursing and medicine that might change the way self-compassion is viewed or experienced as well as whether it buffers stress’s effects [59]. Universities could set up positive education for undergraduate nursing students, such as compassion cultivation training (CCT) program [60], self-care education, and resilience intervention [61], promoting self-compassion through nurturing kindness, acceptance, or mindfulness and meditation skills toward the self can aid in the development of a positive view of the self to refer to during negative experiences [15, 62].

### Implications

The constructivist grounded theory with the life grid approach was employed in the present study to define the theoretical model of thriving psychological well-being for nursing students. As described by the grounded theory developed in this study, psychological well-being promotion for undergraduate nursing students is embodied by two primary categories of behavior patterns. Associated with each positive behavior are derived through healthy social interaction and self-related aspects and phenomena. The transition to nursing identity can be difficult; however, the findings provide practical guidance for undergraduate nursing students, nursing educators, and policy-makers. Therefore, increasing awareness of these specific pathways may lead to greater psychological well-being and should be integrated into interventions.

### Limitations

It is also important to acknowledge several methodological limitations to the current study. Firstly, the heterogeneity of the study sample was limited in terms of demographics as the participants were selected from only three universities in China. Secondly, the data in the present study were obtained from reconstructions of experiences, which may produce recall bias. Finally, the language used in the interviews was Mandarin while the paper was written is in English. An impoverishment may remain within the participant quotes despite every effort in translation.

### Future directions

Certain potential study directions are recommended based on the present findings. To begin with, research should include a more diversified sample of nursing students from various institutions, enrollment statuses, degrees, and financing sources. Moreover, future research could investigate the cultural and social aspects that may benefit or impede nursing students' experiences with PWB. Finally, longitudinal studies should collect real-time PWB data from undergraduate nursing students throughout their journey.

## Conclusion

The constructivist grounded theory study offers to ingratiate the theoretical model to promote undergraduate nursing students' PWB. Participants generally have experienced both peaks and troughs in their PWB. The psychological counseling and support for nursing students during their clinical placement, the period just entered the university time as well as after the repeated COVID-19 outbreak need to be enhanced. These holistic and multifaceted sets of supportive elements based on a positive psychology perspective are essential to enhance the psychological well-being of nursing students. Nursing educators and administrators should develop appropriate educational programs and interventions based on the findings of the present study to cope with the demand.

## Data Availability

The datasets generated and/or analyzed during the current study are not publicly available due to protecting the confidentiality of the participants but are available from the corresponding author upon reasonable request.
